# Deletion of PTH Rescues Skeletal Abnormalities and High Osteopontin Levels in *Klotho* ^−/−^ Mice

**DOI:** 10.1371/journal.pgen.1002726

**Published:** 2012-05-17

**Authors:** Quan Yuan, Tadatoshi Sato, Michael Densmore, Hiroaki Saito, Christiane Schüler, Reinhold G. Erben, Beate Lanske

**Affiliations:** 1 Department of Developmental Biology, Harvard School of Dental Medicine, Boston, Massachusetts, United States of America; 2 State Key Laboratory of Oral Diseases, Sichuan University, Chengdu, China; 3 Department of Oral Medicine, Infection, and Immunity, Harvard School of Dental Medicine, Boston, Massachusetts, United States of America; 4 Institute of Physiology, Pathophysiology, and Biophysics, Department of Biomedical Sciences, University of Veterinary Medicine, Vienna, Austria; Indiana University School of Medicine, United States of America

## Abstract

Maintenance of normal mineral ion homeostasis is crucial for many biological activities, including proper mineralization of the skeleton. Parathyroid hormone (PTH), Klotho, and FGF23 have been shown to act as key regulators of serum calcium and phosphate homeostasis through a complex feedback mechanism. The phenotypes of *Fgf23^−/−^* and *Klotho^−/−^* (*Kl^−/−^*) mice are very similar and include hypercalcemia, hyperphosphatemia, hypervitaminosis D, suppressed PTH levels, and severe osteomalacia/osteoidosis. We recently reported that complete ablation of PTH from *Fgf23^−/−^* mice ameliorated the phenotype in *Fgf23^−/−^/PTH^−/−^* mice by suppressing serum vitamin D and calcium levels. The severe osteomalacia in *Fgf23^−/−^* mice, however, persisted, suggesting that a different mechanism is responsible for this mineralization defect. In the current study, we demonstrate that deletion of PTH from *Kl^−/−^* (*Kl^−/−^/PTH^−/−^* or *DKO*) mice corrects the abnormal skeletal phenotype. Bone turnover markers are restored to wild-type levels; and, more importantly, the skeletal mineralization defect is completely rescued in *Kl^−/−^/PTH^−/−^* mice. Interestingly, the correction of the osteomalacia is accompanied by a reduction in the high levels of osteopontin (Opn) in bone and serum. Such a reduction in Opn levels could not be observed in *Fgf23^−/−^/PTH^−/−^* mice, and these mice showed sustained osteomalacia. This significant *in vivo* finding is corroborated by *in vitro* studies using calvarial osteoblast cultures that show normalized Opn expression and rescued mineralization in *Kl^−/−^/PTH^−/−^* mice. Moreover, continuous PTH infusion of *Kl^−/−^* mice significantly increased Opn levels and osteoid volume, and decreased trabecular bone volume. In summary, our results demonstrate for the first time that PTH directly impacts the mineralization disorders and skeletal deformities of *Kl^−/−^*, but not of *Fgf23^−/−^* mice, possibly by regulating Opn expression. These are significant new perceptions into the role of PTH in skeletal and disease processes and suggest FGF23-independent interactions of PTH with Klotho.

## Author Summary

Maintenance of normal mineral ion homeostasis is crucial for many biological activities, including proper mineralization of the skeleton. PTH, Klotho, and FGF23 are the key regulators of blood mineral ion homeostasis. Klotho is a type-I membrane protein and has been identified as cofactor required for FGF23 to bind and activate its receptor. Loss of either Klotho or Fgf23 activity results in a similar abnormal phenotype, including severe defects in skeletal mineralization and alterations in mineral ion balance. Here we describe a new mouse model in which we eliminated PTH from *Kl^−/−^* mice, and we can show that the skeletal mineralization defect was completely rescued in *Kl^−/−^*/*PTH^−/−^* mice and that this phenomenon was accompanied by a reduction in the high levels of osteopontin in bone and serum. We also present additional data showing that continuous infusion of *Kl^−/−^* mice with PTH results in an elevation in Opn levels and subsequently increased osteoid volume. Interestingly, this result differs from our previous report in which we describe that the osteomalacia and the high Opn levels in *Fgf23^−/−^*/*PTH^−/−^* mice persisted. Our finding suggests that PTH, possibly by regulating osteopontin, is responsible for the skeletal mineralization defect in *Kl^−/−^* mice, but not in *Fgf23^−/−^* mice.

## Introduction

Maintaining normal mineral ion homeostasis is crucial for essential biological activities that include but are not limited to energy metabolism, signaling activities, and normal skeletal growth, development and function. Blood calcium and phosphate levels are determined by counterbalance between absorption from the intestine, mobilization from bone and excretion from the kidney into urine [1]. This complex process is regulated by several endocrine factors, including parathyroid hormone (PTH), FGF23 and active Vitamin D, which have been widely studied [2]–[5]. More recently, another protein, Klotho, has been suggested to have an important role in regulating calcium and phosphate homeostasis.

Klotho is a type-I membrane protein mainly expressed in kidneys, parathyroid glands, and the choroid plexus [6]. It is also related to β-glucosidases and is found in a soluble form in blood and cerebrospinal fluid [7], [8]. Klotho forms a complex with the FGF receptor 1c (FGFR1c), thereby converting this canonical FGF receptor into a receptor specific for FGF23 [9], a negative regulator of serum phosphate. FGF23 uses the FGFR1c/Klotho complex to directly target the kidney where it induces phosphate wasting by decreasing the expression of the sodium-dependent phosphate co-transporters NaPi2a and NaPi2c [10], [11]. Klotho also regulates serum calcium by affecting both parathyroid gland and kidney independent of FGF23. When serum calcium is low, Klotho hydrolyzes extracellular sugar residues on the renal transepithelial calcium channel TRPV5, entrapping the channel in the plasma membrane [12]. This maintains continuous calcium channel activity and membrane calcium permeability, leading to an increase in tubular reabsorption of calcium in the kidneys and finally increased serum calcium. In the parathyroid gland, Klotho recruits Na^+^/K^+^-ATPase to the cell surface, which results in an increase in PTH production, which in turn elevates serum calcium level [13], [14]. In addition to Klotho's independent effect on PTH induction, it can act together with FGF23 to decrease PTH levels [15], [16]. An *in vivo* study has shown that injection of FGF23 protein into rats can lower PTH secretion and expression [15]. This was confirmed by *in vitro* experiments using parathyroid gland cultures [16].

The function of Klotho as a cofactor of FGF23 was confirmed in studies by us and others showing that genetic ablation of either *Fgf23* or *Klotho* results in a similar phenotype [17]–[19]. Both *Klotho* knockout (*Kl^−/−^*) and *Fgf23* knockout (*Fgf23^−/−^*) mice exhibit hypercalcemia, hyperphosphatemia with low to undetectable PTH levels [20]–[22], and severe osteomalacia. We have previously described [23] that deletion of PTH in *Fgf23^−/−^* mice ameliorated the abnormal phenotype by normalizing serum Ca^2+^ and lowering serum vitamin D levels, however, the severe osteomalacia persisted in *Fgf23^−/−^/PTH^−/−^* mice. Because Klotho also has FGF23-independent functions, we thought it would be important to investigate the effects of deleting PTH from *Kl^−/−^* mice. We demonstrate that the skeletal mineralization defect in *Kl^−/−^/PTH^−/−^* mice was completely rescued and that this phenomenon was accompanied by a reduction in the high levels of osteopontin in bone and serum, a finding that could not be observed in *Fgf23^−/−^/PTH^−/−^* mice. We also present data showing that continuous infusion of *Kl^−/−^* mice with PTH results in an elevation in OPN levels and subsequently increased osteoid volume. Our finding demonstrates for the first time that the skeletal abnormalities and the bone mineralization defect in *Kl^−/−^* can be rescued by ablation of PTH actions. Our data suggest regulatory actions on osteopontin by PTH, an important observation with clinical significance. The identical levels in serum calcium, phosphate and vitamin D in *Fgf23^−/−^/PTH^−/−^* and *Kl^−/−^/PTH^−/−^* preclude any effects of these parameters on the regulation of skeletal mineralization and/or Opn levels in these mice. Additional studies are required to identify the mechanisms by which PTH affects osteopontin and mineralization in *Kl^−/−^* but not in *Fgf23^−/−^*mice.

## Results

### Deletion of PTH results in healthier *Kl^−/−^*/*PTH^−/−^* double-knockout animals

**Figure 1:**
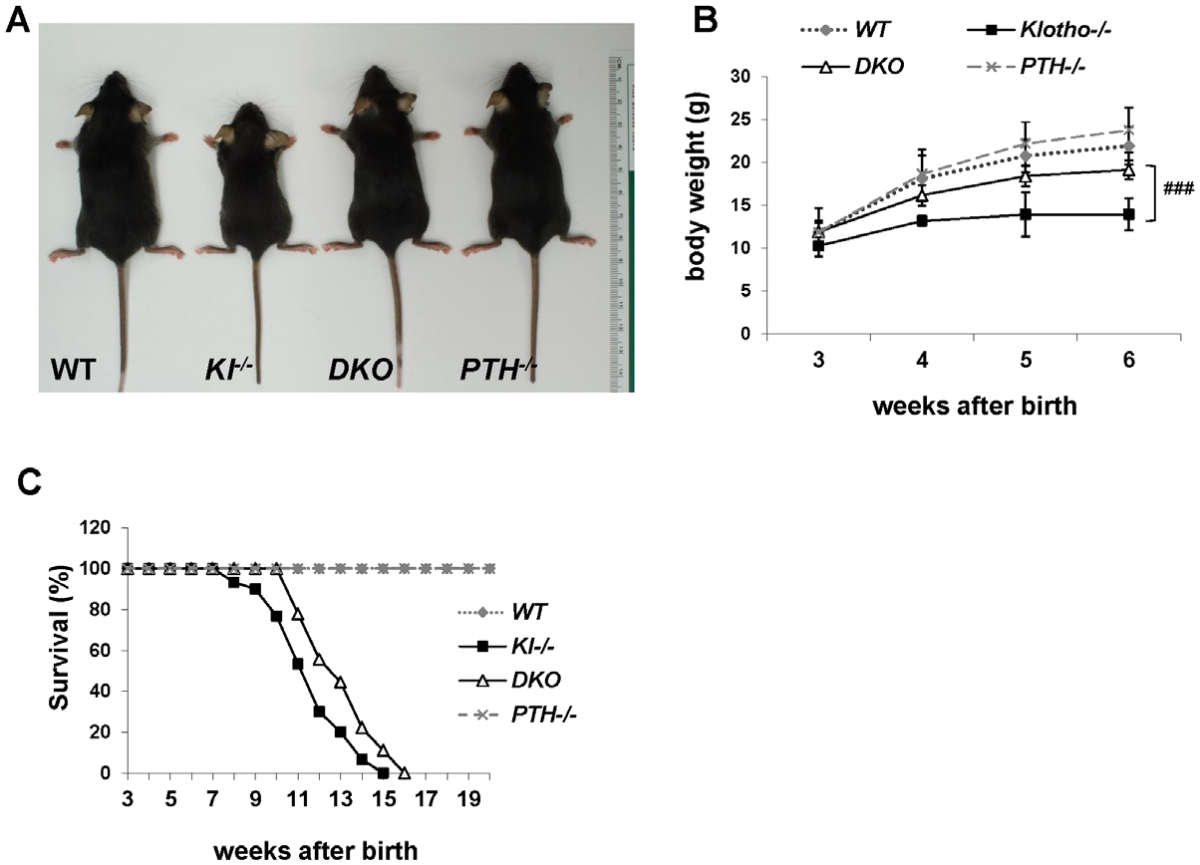
Macroscopic phenotype of 6-wk-old mice. (A) Overall phenotype of littermates. Complete deletion of *PTH* from *Kl^−/−^* mice resulted in larger, heavier, and more active *DKO* mice compared to *Kl^−/−^* littermates. The body weight of *DKO* mice is significantly higher than that of *Kl^−/−^* mice (B), and the lifespan is slightly improved (C). ###: *p*<0.001 *vs Kl^−/−^*.

We successfully generated *Kl^−/−^*/*PTH^−/−^* (*DKO*) mice by interbreeding heterozygous *Kl^+/−^* and *PTH^+/−^* mice. *DKO* mice were more active, healthier and larger in size than *Kl^−/−^* mice and more comparable to wild-type and *PTH^−/−^* single knock-out littermates (Figure 1A). *DKO* mice did not show any obvious gross abnormalities with regard to movement and physical activities, whereas *Kl^−/−^* littermates were severely weakened, showing restricted movement as well as sluggish physical activities. *DKO* mouse body weight was significantly higher than that of *Kl^−/−^* mice (Figure 1B). Compared to the *Kl^−/−^* mice, *DKO* mice also showed a clear improvement in life span as evidenced by a right shift of the survival curve (Figure 1C). All mice, however, died before 16 weeks of age, probably due to the severe soft tissue calcifications such as found in kidney and lung of both *Kl^−/−^* and *DKO* mice (Figure S1).

### Serum biochemistry

**Figure 2:**
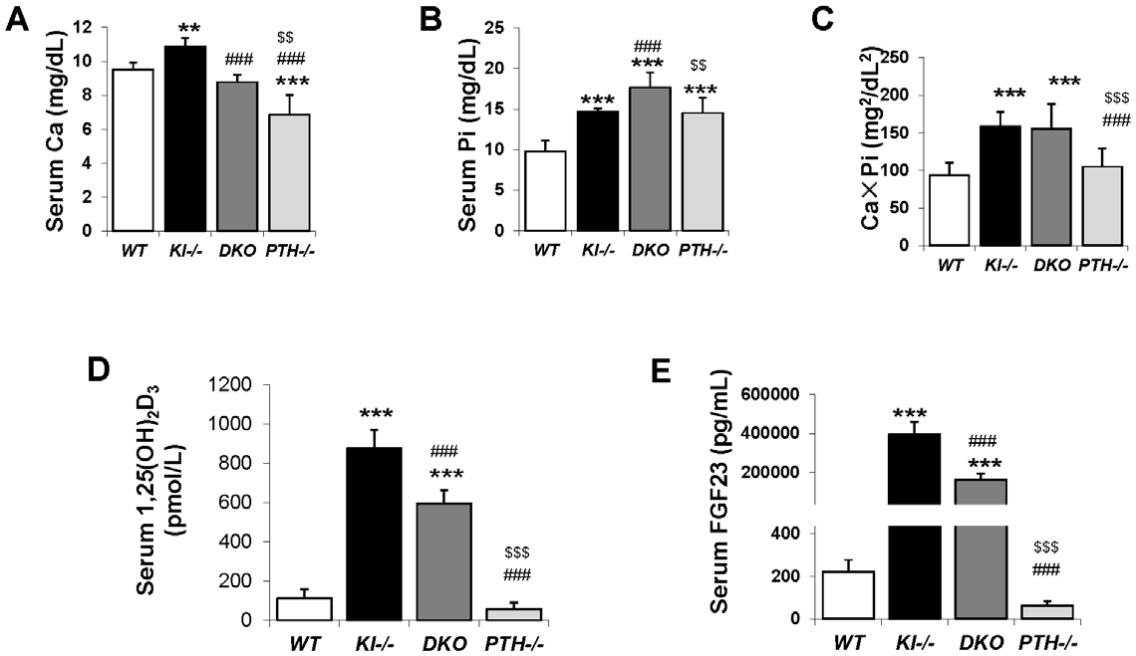
Serum biochemical measurement. Abnormal serum calcium levels observed in *Kl^−/−^* and *PTH^−/−^* mice were normalized in *DKO* mice (A). As for phosphate, *DKO* mice showed a further increase over already high levels in *Kl^−/−^* and *PTH^−/−^* mice (B). *Kl^−/−^* and DKO mice showed similarly high CaPi product (C). The serum levels of intact FGF23 (D) and 1,25(OH)_2_D (E) in *DKO* mice were partially reduced compared to that of *Kl^−/−^* mice. *: *p*<0.05, **: *p*<0.01, ***: *p*<0.001*vs WT*; ###: *p*<0.001*vs Kl^−/−^*; and $$: *p*<0.01, $$$: *p*<0.001 *vs DKO*.

Six-week old *Kl^−/−^* and *PTH^−/−^* mice were severely hypercalcemic (10.86±0.52 mg/dL) and hypocalcemic (6.85±1.17 mg/dL), respectively. However *DKO* animals were normocalcemic (8.78±0.42 mg/dL) at 6 weeks, comparable to *WT* control animals (9.52±0.41 mg/dL), (Figure 2A). Serum phosphate levels in both *Kl^−/−^* (14.61±0.48 mg/dL) and *PTH^−/−^* (14.52±1.87 mg/dL) mice were significantly higher compared to those in *WT* mice (9.75±1.34 mg/dL), (Figure 2B). Interestingly, *DKO* exhibited a further increase in serum phosphate to levels (17.65±1.86 mg/dL) far exceeding those in single *Kl^−/−^* or *PTH^−/−^* mice. We determined the total mineral content by calculating the calcium/phosphate product and found that *Kl^−/−^* and *DKO* mice exhibited similarly high levels (Figure 2C). Measurements of serum 1,25(OH)_2_D levels showed increased amounts in *Kl^−/−^* single knockout mice compared to those in *WT* and *PTH^−/−^* mice. Serum 1,25(OH)_2_D levels in *DKO* mice were significantly reduced compared to those of *Kl^−/−^* single knockout mice, but were still significantly higher than in wild-type or *PTH^−/−^* mice (Figure 2D). We also measured intact serum FGF23 levels and found that *DKO* mice had a 50% decrease in serum FGF23 compared to *Kl^−/−^* mice, however the levels were still significantly higher (1000 fold) than those in wild-type or *PTH^−/−^* mice (Figure 2E).

### Deletion of *PTH* rescues skeletal abnormalities of *Kl^−/−^* mice

**Figure 3:**
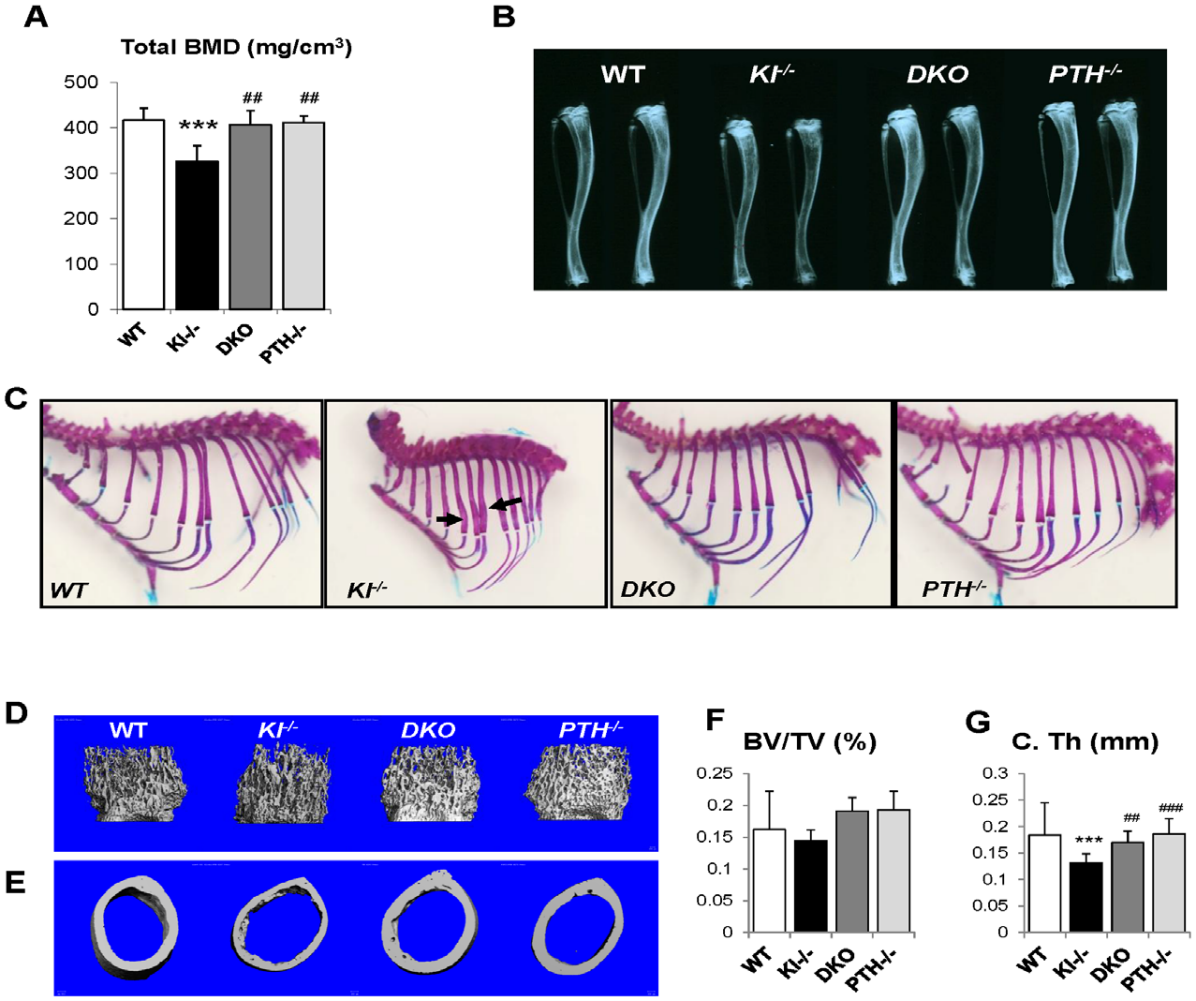
Rescued skeletal phenotype. Bone Mineral Density (BMD) of distal ends of femurs from 6-week-old mice (A). Radiographs of the tibiae from all genotypes at 6 wk of age indicate that length and radiopacity of the tibiae were restored in *DKO* mice (B). Alizarin red S and Alcian blue staining shows that the abnormally wide ribs in *Kl^−/−^* mice (indicated by black arrow) were not observed in the *DKO* mice (C). Representative microCT images of distal femoral metaphyses (D) and midshaft cortical bone (E) and quantitative analysis (F, G). The midshaft cortical thickness (C.Th) of the *DKO* mice was restored to a volume comparable to that of *WT* and *PTH^−/−^* mice. ***: *p*<0.001*vs WT*; ##: *p*<0.01, ###: *p*<0.001 *vs Kl^−/−^*.

We performed peripheral quantitative computerized tomography (pQCT) to analyze the bone density in the femurs of all genotypes. *Kl^−/−^* mice showed decreased total BMD in the distal femur metaphysis (Figure 3A). Ablation of the *PTH* gene from these mice significantly increased the BMD, which was now comparable to that of *WT* and *PTH^−/−^* mice.

Radiographs showed that the length and radiopacity of the tibiae from *DKO* mice were increased and comparable to those of *WT* and *PTH^−/−^* mice (Figure 3B). We performed Alizarin red S and Alcian blue staining to determine the mineralization pattern of the bones. As shown in Figure 3C, *Kl^−/−^* mice exhibited abnormally widened ribs, but this abnormality could not be observed in the *DKO* mice, suggesting an improved skeletal architecture.

MicroCT (μCT) analysis of the femurs was performed on all four genotypes. Representative images of distal femoral metaphyses and midshaft cortex are shown in Figure 3D and 3E. Quantification of trabecular bone volume fraction demonstrated that there is no significant difference between each genotype (Figure 3F). As μCT can only detect mineralized bone, *Kl^−/−^* mice didn't show increased trabecular volume. However, the midshaft cortical thickness of the *Kl^−/−^* mice (0.132±0.013 mm) was significantly reduced compared to the other groups (Figure 3G). It was restored in *DKO* mice (0.170±0.015 mm) to a volume comparable to that in *WT* (0.184±0.011 mm) and *PTH^−/−^* (0.186±0.016 mm) mice (Figure 3G).

**Figure 4:**
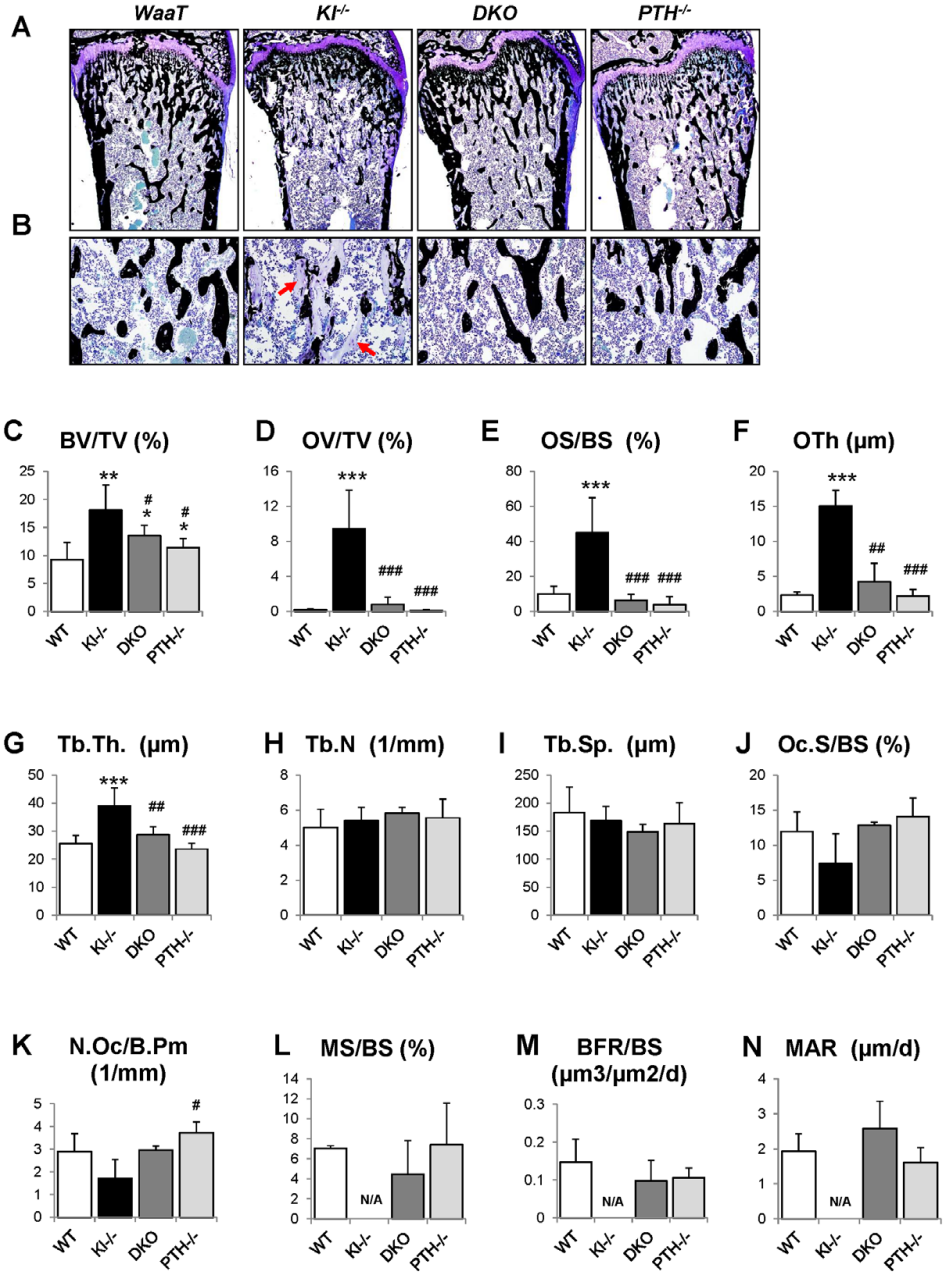
Histological and histomorphometric analyses. Undecalcified sections of distal ends of femurs from 6wk-old littermates were stained with von Kossa and McNeal (A, B). High magnification of the secondary spongiosa shows heavily unmineralized osteoid in *Kl^−/−^* mice but not in *DKO* mice (B). Histomorphometric analysis (C–N) confirmed that the skeletal architecture and mineralization defect of *Kl^−/−^* mice were rescued in *DKO* mice. BV/TV: bone volumes; OV/TV: osteoid volume; OS/BS: osteoid surface/bone surface; OTh; osteoid thickness; Tb.Th: trabecular thickness; Tb.N: trabecular number; Tb.Sp: trabecular separation; Oc.S/BS: osteoclast surface/bone surface; N.Oc/B.Pm: osteoclast number/bone perimeter; MS/BS: mineral surface/bone surface; BFR: bone formation rate, and MAR: mineral apposition rate. *: *p*<0.05, **: *p*<0.01, ***: *p*<0.001*vs WT*; and #: *p*<0.05, ##: *p*<0.01, ###: *p*<0.001 *vs Kl^−/−^*.

We further analyzed the skeletal properties by generating undecalcified methylmethacrylate sections from the distal ends of femurs to confirm the observed improvement in bone mass compared to *Kl^−/−^* mice (Figure 4A and 4B). Most importantly, the severe osteoidosis seen in the secondary spongiosa of *Kl^−/−^* mice was completely absent in *DKO* mice (Figure 4B), indicating the mineralization defect of *Kl^−/−^* mice was rescued by *PTH* ablation. To quantify this observation, we performed histomorphometric analyses (Figure 4C–4N). The increased trabecular bone volume observed in *Kl^−/−^* mice (18.1±4.4) was restored in *DKO* mice (13.5±1.9) to values close to those of *PTH^−/−^* mice (11.4±1.6). Interestingly, deletion of *PTH* also rescued the severe mineralization defect of *Kl^−/−^* mice as evidenced by normalized osteoid volume (OV/TV), osteoid surface (OS/BS) and thickness (OTh) in *DKO* mice (Figure 4D–4F). *DKO* mice also showed normal trabecular thickness (Tb.Th.), trabecular number (Tb.N.) and separation (Tb.Sp.), as well as osteoclast surface (Oc.S/BS) and osteoclast numbers (N.Oc/B.Pm) (Figure 4G–4K). We also found that the dynamic parameters, including mineral surface (MS/BS), bone formation rate (BFR/BS) and mineral apposition rate (MAR) (Figure 4L–4N), were normalized in *DKO* mice while the bone labeling in *Kl^−/−^* mice was unsuccessful due to the severe mineralization defect.

**Figure 5:**
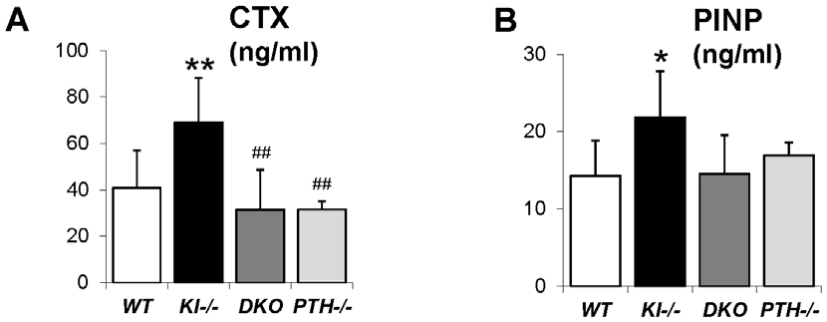
Measurement of serum CTX and PINP. Measurement of serum CTX (A) and PINP (B), indicating normalized bone turnover in *DKO* mice. *: *p*<0.05, **: *p*<0.01*vs WT;* ##: *p*<0.01*vs Kl^−/−^*.

We next analyzed the concentration of serum markers for bone turnover. Consistent with histomorphometric data, serum levels of the carboxyl-terminal telopeptide of type 1 collagen (CTX), a biomarker of bone resorption activity, were comparable in *DKO* (31.5±17.2 ng/ml), *WT* (40.8.5±16.2 ng/ml) and *PTH^−/−^* (31.5±3.6 ng/ml) mice (Figure 5A). Similarly, circulating levels of N-terminal propeptide of type I procollagen (PINP), a reliable and sensitive marker of bone formation, were significantly elevated in *Kl^−/−^* mice (21.8±6.0 ng/ml) (Figure 5B). PINP levels were restored in *DKO* mice (14.5±5.0 ng/ml) to levels observed in *WT* (14.2±4.6 ng/ml) and *PTH^−/−^* (17.0±1.6 ng/ml).

### Rescued bone mineralization is accompanied by normalized expression of Opn in *DKO* mice

**Figure 6:**
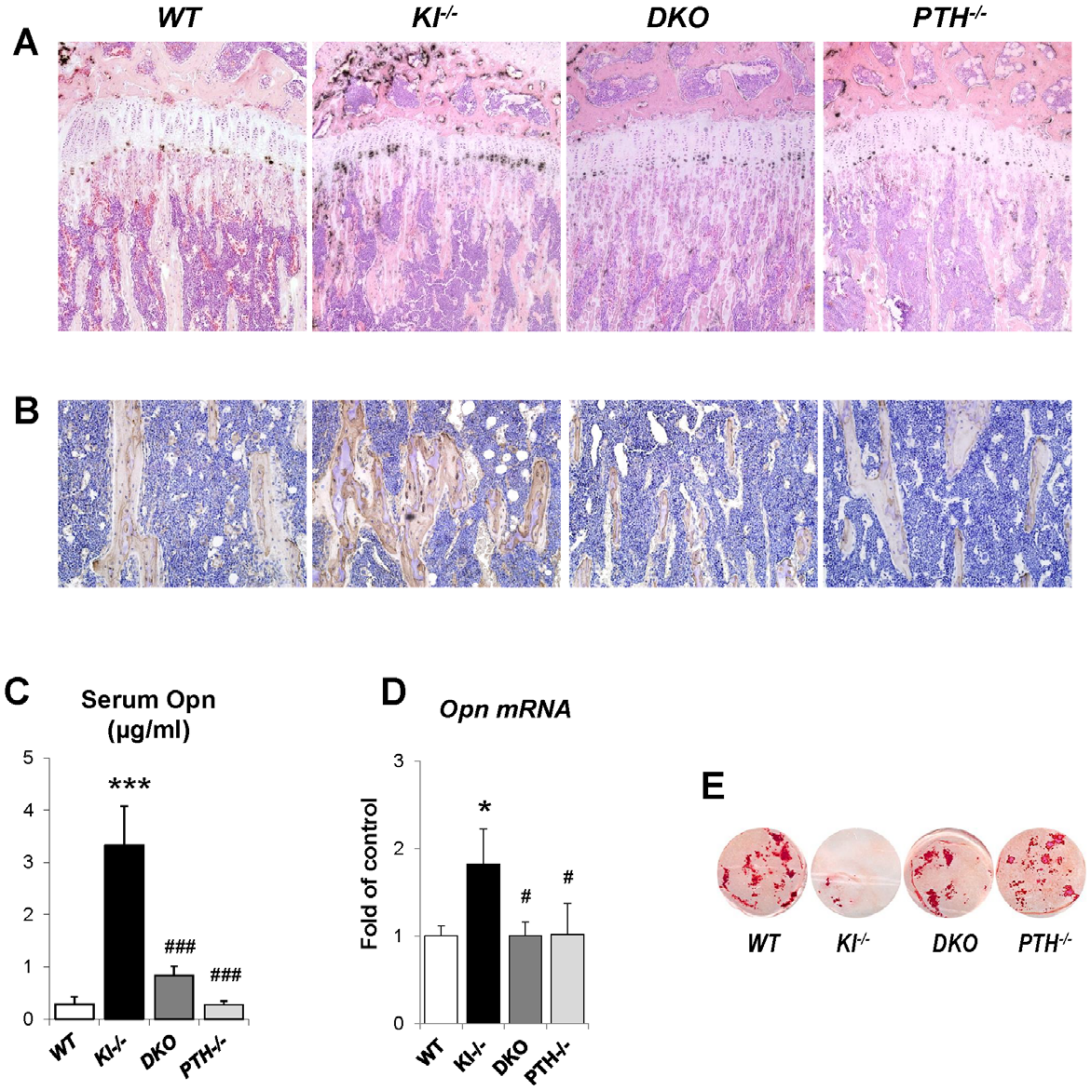
Normalized expression of Opn in *DKO* mice. In situ hybridization (A) and immunohistochemical staining (B) on paraffin sections prepared from tibiae of animals at 6 weeks of age. The elevated *Opn* expression in *Kl^−/−^* mice was normalized in *DKO* mice. (C) Measurements of the serum Opn levels. Quantitive PCR confirmed the normal expression of *Opn* in the osteoblasts isolated from *DKO* mice (D). *DKO* osteoblasts also exhibited normal mineralization as evaluated by Alizarin red staining (E). *: *p*<0.05, ***: *p*<0.001*vs WT*; and #: *p*<0.05, ###: *p*<0.001 *vs Kl^−/−^*.

To explain the rescue in bone mineralization in *DKO* mice, we compared the expression of osteopontin and other factors associated with skeletal mineralization in all genotypes. As shown by *in situ* hybridization and immunohistochemical staining on decalcified paraffin sections (Figure 6A and 6B), the expression of Opn, an inhibitor of osteogenic mineralization and member of the SIBLING protein family, is abnormally high in the bone. Interestingly, its expression was normalized in *DKO* mice to levels seen in *WT* and *PTH^−/−^* mice (Figure 6A and 6B). We also measured serum Opn levels using an ELISA kit and were able to confirm significantly elevated serum Opn levels in *Kl^−/−^* mice, which were restored to normal levels in *DKO* mice (Figure 6C). Since we previously reported increased Opn expression in *Fgf23^−/−^* bones [17], we were interested in also examining their serum Opn levels and found that they were also significantly increased. In contrast, however, deletion of *PTH* from *Fgf23^−/−^* mice failed to normalize their serum Opn levels (Figure S2).

To further confirm the *in vivo* observations in *Kl^−/−^* and *DKO* mice calvarial osteoblasts from 2-day-old littermates were isolated. Cells were cultured in osteogenic medium for 2 weeks and RNA was isolated. qPCR analyses showed normal expression of *Opn* in osteoblasts of *DKO* mice (Figure 6D). These osteoblasts also exhibited normal mineralization as evaluated by Alizarin red staining while the mineralization of the osteoblasts from *Kl^−/−^* mice was markedly impaired (Figure 6E). We also evaluated the expression of Dmp1 and Matrix gla protein (Mgp) by *in situ* hybridization and/or qPCR. Both were significantly increased in *Kl^−/−^* mice, but rescued in *DKO* mice (Figure S3).

### Infusion of PTH increases Opn levels and led to more severe mineralization defect

**Figure 7:**
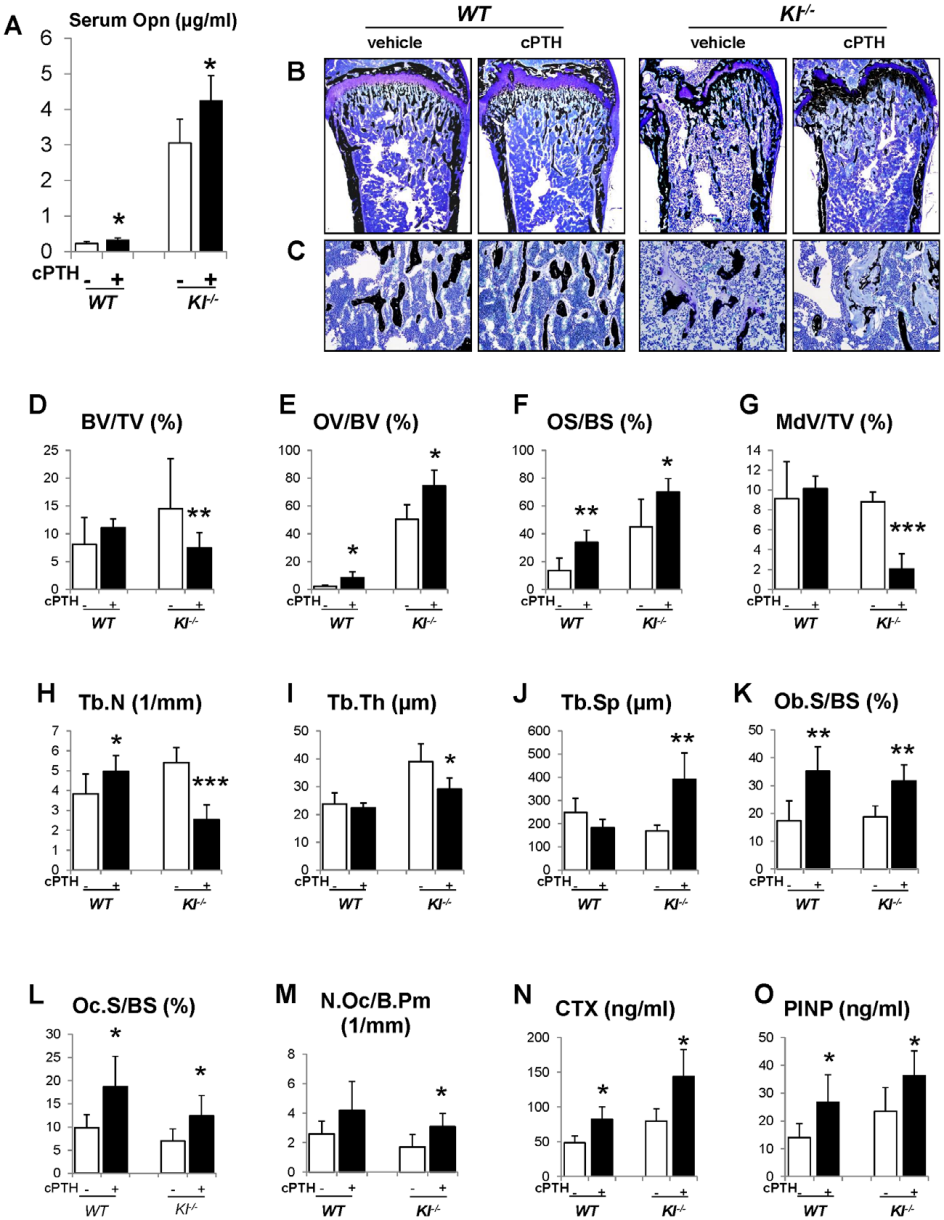
Infusion of PTH increases Opn levels and leads to a more severe mineralization defect. PTH infusion increased serum Opn levels (A). Undecalcified sections of distal ends of femurs were stained with von Kossa and McNeal (B, C). Histomorphometric analysis (D–M) showed that PTH infusion decreased the bone volume (BV/TV) and increased osteoid volume (OV/BV) in *Kl^−/−^* mice. Serum CTX (N) and PINP (O) levels were significantly increased after PTH infusion. MdV/TV: mineralized bone volume/total volume. Ob.S/BS: osteoblast surface/bone surface. *: *p*<0.05, **: *p*<0.01, ***: *p*<0.001*vs* vehicle controls.

To further investigate the role of Opn in regulating the mineralization, we perfused PTH (1–34) peptides into the *WT* and *Kl^−/−^* mice using osmotic minipumps. After continuous infusion for 3 weeks, we observed that the serum Opn levels were significantly elevated in both *WT* and *Kl^−/−^* mice (Figure 7A). As expected, serum phosphate levels were significantly decreased in both kinds of mice (Figure S4). PTH infusion, as shown in Figure 7B–7D, did not change the bone volume in *WT* mice, but significantly decreased it in *Kl^−/−^* mice. More importantly, OV/BV (Figure 7E) and OS/BS (Figure 7F) of *Kl^−/−^* mice were further increased by the PTH infusion, while the mineralized bone volume (MdV/TV) (Figure 7G) was significantly decreased, indicating that extraneous PTH induces Opn and thereby worsens the skeletal mineralization defect in the *Kl^−/−^* mice. Continuous PTH infusion also increased osteoblast surface (Ob.S/BS), Oc.S/BS and N.Oc/B.Pm (Figure 7K–7M) in both *WT* and *Kl^−/−^* mice. Opn contains a RGD sequence, which is important for osteoclast attachment on the bone. Thus it is not surprising that infusion of PTH resulted in a significant elevation in serum CTX levels, indicating increased osteoclastic resorption (Figure 7N). In addition, PTH infusion significantly elevated serum PINP levels in both *WT* and *Kl^−/−^* mice (Figure 7O).

## Discussion

This is the first study using a genetic mouse model with dual ablation of the *Klotho* and *PTH* genes. The results show that deletion of *PTH* from *Kl^−/−^* mice resulted in healthier mice with normalization of serum calcium levels and complete rescue of the skeletal phenotype, suggesting that PTH is a crucial contributor to the skeletal abnormalities caused by loss of Klotho function. More importantly, we found that deletion of the *PTH* completely rescued the mineralization defect in *Kl^−/−^* mice. *Kl^−/−^* mice, as well as *Fgf23^−/−^* mice, exhibit a severe mineralization defect in their bones despite excesses in serum calcium and phosphate when compared to *WT* mice. The underlying reason for this is largely unknown but FGF23 is recognized as an inhibitor of mineralization. Serum Fgf23 levels in *Kl^−/−^* mice are two thousand fold higher than in wild-type littermates. Wang *et al* [24] show that adenoviral overexpression of FGF23 in rat calvarial cells inhibits bone mineralization independent of its systemic effects on phosphate homeostasis. Our previous report also demonstrated that FGF23 treatment of primary calvarial osteoblasts from wild-type mice leads to an inhibition of mineralization [18]. However, FGF23 requires Klotho for its actions [9], [20], [25]. In the absence of Klotho, FGF23 has very low affinity to the FGFR1 and cannot induce signal transduction (phosphorylation) [9], [25], [26].

Another intriguing possibility is that Klotho may have a specific function in osteoblasts associated with PTH. Although Klotho has been widely accepted as the cofactor of FGF23 signaling [9], [26], [27], and *Kl^−/−^* and *Fgf23^−/−^* mice share very similar phenotypes [20], we show here that deletion of *PTH* fully rescues the mineralization defect in *Kl^−/−^* mice, but could not improve this defect in *Fgf23^−/−^* animals [23]. More recently, Klotho has been reported to be expressed in the osteoblastic cell linage [28]. In addition, previous studies have shown that Klotho does exert endogenous actions in calcium homeostasis and control of PTH secretion [13], [14] that are independent of FGF23. Similarly, Klotho directly mediates secretion of PTH through recruitment of Na^+^/K^+^ ATPase to the plasma membrane [14]. Recent studies also showed that *Kl^−/−^* mice have increased trabecular bone [29], [30]. In this study, we confirmed elevated bone volume in *Kl^−/−^* mice compared to the normal bone volume in *Fgf23^−/−^* mice. More importantly, cultured osteoblasts isolated from *Kl^−/−^* pups at the age of 2-days showed markedly impaired mineralization, suggesting that Klotho may play a specific role in osteoblasts. Moreover, we detected low Klotho expression by qPCR in both cultured osteoblasts and isolated cortical bone (Figure S5). An osteoblast-specific *Klotho* knockout mouse model may be required to dissect the role of Klotho during skeletogenesis.

To examine the bone mineralization defect in more detail, we determined the expression of Opn by *in situ* hybridization, immunohistochemistry, ELISA and quantitative PCR. Opn is a well-known mineralization inhibitor, and mice deficient in Opn show soft tissue calcification and premature bone mineralization [31], [32]. Our analyses showed that the increased amount of Opn detected in *Kl^−/−^* mice was normalized in *DKO* mice. PTH is known to be an important regulator of Opn and can induce the expression of *Opn* in MC3T3-E1 cells within 3 hours [33], [34]. Therefore, the complete ablation of PTH might be responsible for the normalization of the increased Opn levels in *Kl^−/−^* mice and subsequently rescue the mineralization defect. Furthermore, infusion of exogenous PTH into *Kl^−/−^* mice resulted in a significant elevation in Opn levels with a worsened mineralization defect. This further strengthens our hypothesis that PTH could regulate skeletal mineralization in *Kl^−/−^* mice via Opn.

Although studies suggest that phosphate could also regulate the expression of Opn [35]–[37], our previous study using *Fgf23^−/−^/NaPi2a^−/−^* mice suggested that the elevated expression of Opn in bones of *Fgf23^−/−^* mice is at least partially independent of systemic phosphate levels [18]. This was further supported by our observation in this study that *PTH^−/−^* and *DKO* mice had normal expression of Opn despite very high serum phosphate levels. In addition, we also found in this study that PTH infusion could increase the serum Opn, even while the serum phosphate levels were decreased (Figure S4). Moreover, serum calcium, phosphate and vitamin D levels in *Fgf23^−/−^/PTH^−/−^* and *Kl^−/−^/PTH^−/−^* are identical and can therefore not contribute to the regulation of skeletal mineralization and/or Opn levels in these mice.

In summary, the findings in this study demonstrate that genetic ablation of *PTH* resulted in healthier *DKO* mice. More importantly, deletion of PTH completely rescued the skeletal abnormalities, including the severe mineralization defect in *Kl^−/−^* mice, and this effect is very likely associated with normalized expression of Opn in *DKO* mice. Interestingly, we previously showed that deletion of *PTH* in *Fgf23^−/−^* mice could not rescue mineralization, implying an independent function of Klotho in bone. This study demonstrates that the activity of the low level of PTH remaining in *Kl^−/−^* mice contributes to the severe mineralization disorder and skeletal abnormalities caused by the loss of Klotho function. Moreover, we show that Klotho affects mineralization independently of its role as a co-factor for FGF23. Further analyses are needed to determine the independent roles of PTH and Klotho in mineral ion homeostasis and skeletal mineralization and their detailed molecular interactions, including those involving OPN.

## Materials and Methods

### Animals

Heterozygous- *Kl^+/−^* and *PTH^+/−^* animals were interbred to attain wild-type (*WT*), *Kl^−/−^*, *Kl^−/−^*/*PTH^−/−^* (double knockout, *DKO*) and *PTH^−/−^* animals for subsequent analyses. Routine PCR was used to genotype various mice as described previously [20], [38]. The total body weight of each mouse was measured weekly starting at 3 weeks after birth. All studies performed were approved by the Institutional Animal Care and Use Committee at the Harvard Medical School.

### Biochemical analyses

Blood was obtained by puncturing the cheek pouch of animals. Total serum calcium and phosphorus levels were determined using Stanbio LiquiColor (Arsenazo III) and LiquiUV kits (Stanbio Laboratory, Boerne, TX), respectively. Serum concentrations of FGF23 and Opn were measured using commercial kits from Kainos Laboratories, Inc., (Tokyo, Japan), and R&D Systems, Inc. (Minneapolis, MN), respectively. The ELISA kits for 1,25(OH)_2_D, PINP and CTX were purchased from IDS (Fountain Hills, AZ).

### Skeletal mineralization and bone mineral density

The mineralization pattern of the skeleton was analyzed by Alizarin red S and Alcian blue staining in 6- week-old mice, as described by McLeod [39]. Femurs of all genotypes at 6 weeks of age were flushed and exposed to X-ray (20 kV, 5 seconds). As described previously [40], bone mineral density (BMD) and μCT analysis were performed by peripheral quantitative computerized tomography (pQCT) and by using a Scanco Medical μCT 35 system (Scanco), respectively.

### Bone histology and histomorphometry

Processing of undecalcified bone specimens and cancellous bone histomorphometry in the distal femoral metaphysis were performed as described [40]. The area within 0.25 mm from the growth plate was excluded from the measurements.

### Soft tissue calcifications

Von Kossa staining was performed using 5 µm paraffin sections of kidneys and lungs isolated from 9-week-old animals.

### *In situ* hybridization

Complementary ^35^S-UTP-labeled riboprobe osteopontin (Opn) and dentin matrix protein 1 (Dmp1) were used for performing *in situ* hybridization on paraffin sections, as described previously [41].

### Immunohistochemistry

Immunohistochemistry was performed using mouse Opn antibody (R&D, Minneapolis, MN) with a working concentration of 0.5 µg/ml overnight at 4°C. Tissue was stained with anti-goat HRP substrate and DAB (Vector, Burlingame, CA), and then counterstained with hematoxylin.

### Mouse calvarial cell culture

Mouse calvarial cell culture was carried out as previously described [18]. Cells were treated with 50 mg/ml ascorbic acid and 10 mM β-glycerophosphate (βGP) to induce matrix mineralization. Total RNA isolation and Alizarin red S staining were performed 14 days after induction.

### Quantitative real-time PCR

Total RNA was from cultured osteoblasts using Trizol reagents (Invitrogen) according to the manufacturer's protocol. For qRT-PCR, cDNA was prepared using QuantiTec reverse transcription kit (Qiagen) and analyzed with SYBR GreenMaster Mix (SABiosciences) in the iCycler (Bio-Rad) using specific primers designed for each targeted gene. Relative expression was calculated using the 2^−ΔΔCt^ method by normalizing with *Gapdh* housekeeping gene expression, and presented as fold increase relative to control.

### *In vivo* continuous PTH (1–34) infusion

50 µg per kilogram of body weight per day of human PTH 1–34 (Polypeptide Group, France) were delivered into 3-week-old animals for a 3-week period using implanted ALZET osmotic minipumps, Model-1004 (DURECT Corporation, Cupertino, CA). Animals of vehicle group were infused with an equal volume of sterile saline.

### Statistics

Statistically significant differences between groups were evaluated by Student's t-test for comparison between two groups or by one-way analysis of variance (ANOVA) followed by Tukey's test for multiple comparisons. And those between vehicle and PTH-infused groups were evaluated by Student's t-test. All values were expressed as mean ± SD. A *p* value of less than 0.05 was considered to be statistically significant.

## Supporting Information

Figure S1 Von Kossa staining of kidney sections (A) and lung sections (B) isolated from 9-week-old animals. Soft tissue calcification was observed in both *Kl^−/−^* and Klotho/PTH double knockout (*DKO*) mice.
(TIF)

Figure S2 Serum osteopontin (Opn) levels in (A) wild-type (WT) and *Fgf23^−/−^* mice at 3 and 6 weeks of age. (B) Comparison of serum Opn levels between WT, *Fgf23^−/−^*, *Fgf23^−/−^*/*PTH^−/−^ (DKO),* and *PTH^−/−^* mice at 6 weeks. ***: *p*<0.001*vs* vehicle controls, ###: *p*<0.001 *vs Fgf23^−/−^*, $$$: *p*<0.001 *vs DKO.*
(TIF)

Figure S3 Gene expression of Dmp1 and Mgp. (A) *in situ* hybridization of bone sections showed that Dmp1 expression was elevated in *Kl^−/−^* mice. (B and C) mRNA expression of Dmp1 and Mgp in osteoblasts was quantified by qPCR analysis. Calvarial osteoblasts were isolated and cultured in osteogenic medium for 2 weeks. *: *p*<0.05, **: *p*<0.01 *vs WT*; ##: *p*<0.01 *vs Kl^−/−^*.
(TIF)

Figure S4 Serum phosphate measurements. PTH infusion significantly decreased serum phosphate levels in both *WT* and *Kl^−/−^* animals. **: *p*<0.01, ***: *p*<0.001*vs* vehicle controls.
(TIF)

Figure S5 Gene expression of Klotho in bone and osteoblasts quantified by qPCR. Klotho expression was detected in the both cultured osteoblasts and cortical bones. However, it was much lower than that observed in the kidney. Tissues from *Kl^−/−^* mice were used as negative controls. ***: *p*<0.001.
(TIF)
