# Supplementary figures and images for: Deletion of PTH Rescues Skeletal Abnormalities and High Osteopontin Levels in Klotho −/− Mice
Source: PLoS Genet. 2012 May 17;8(5):e1002726. doi: 10.1371/journal.pgen.1002726 (PMC3355080; doi:10.1371/journal.pgen.1002726)

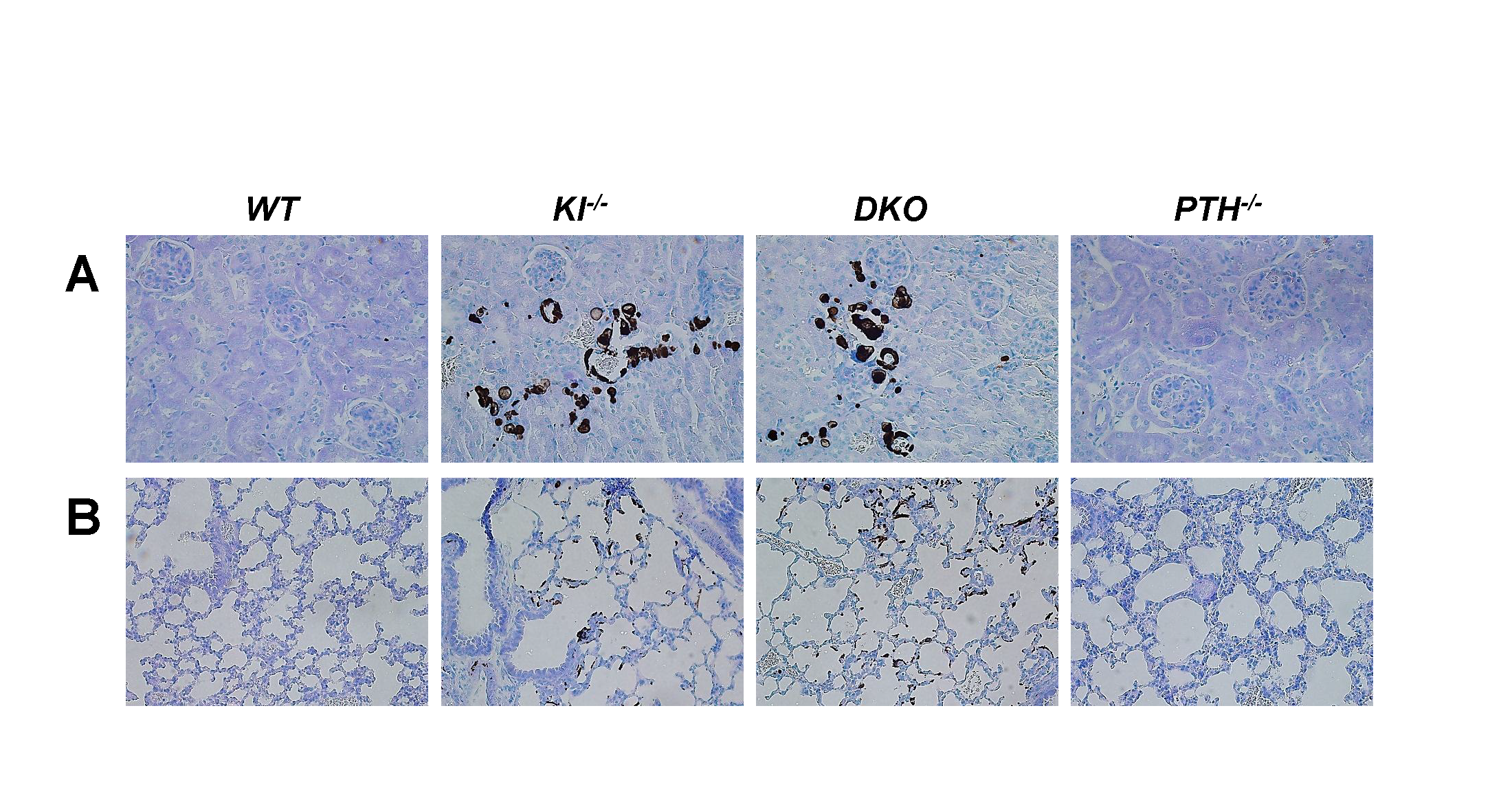

Supplement: Figure S1 — Von Kossa staining of kidney sections (A) and lung sections (B) isolated from 9-week-old animals. Soft tissue calcification was observed in both Kl−/− and Klotho/PTH double knockout (DKO) mice. (TIF) [file pgen.1002726.s001.tif]

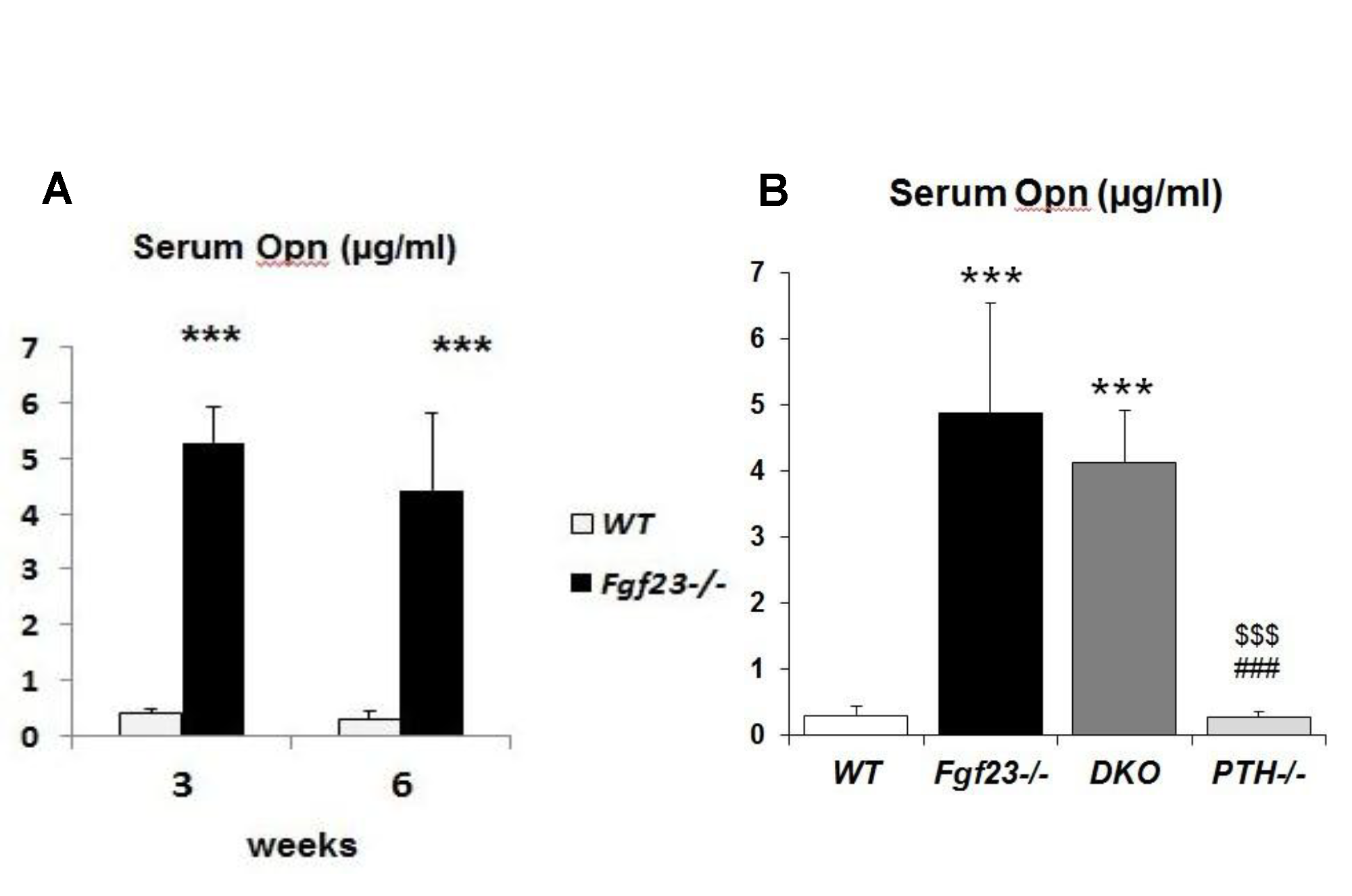

Supplement: Figure S2 — Serum osteopontin (Opn) levels in (A) wild-type (WT) and Fgf23−/− mice at 3 and 6 weeks of age. (B) Comparison of serum Opn levels between WT, Fgf23−/−, Fgf23−/−/PTH−/− (DKO), and PTH−/− mice at 6 weeks. ***: p<0.001vs vehicle controls, ###: p<0.001 vs Fgf23−/−, $$$: p<0.001 vs DKO. (TIF) [file pgen.1002726.s002.tif]

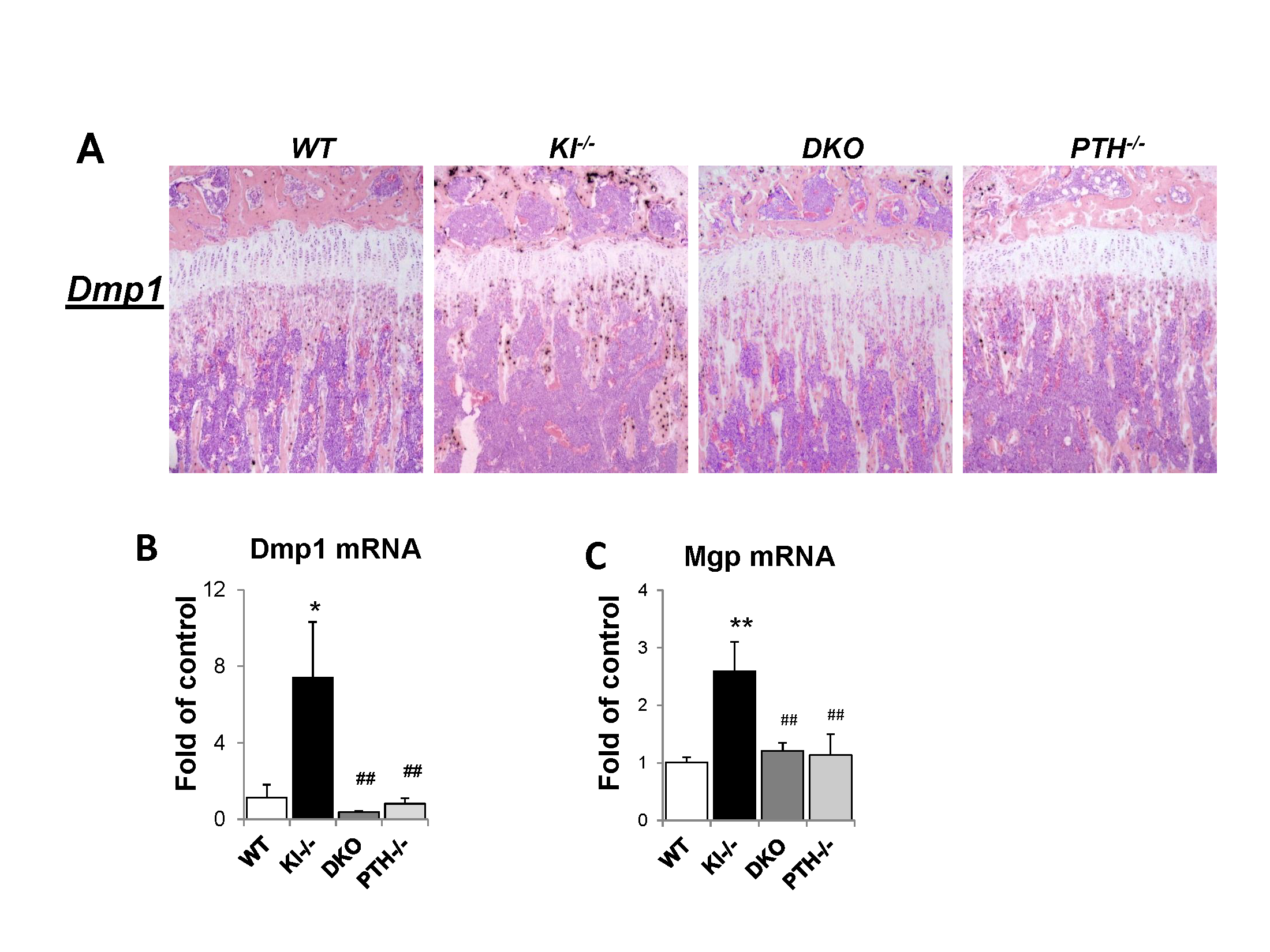

Supplement: Figure S3 — Gene expression of Dmp1 and Mgp. (A) in situ hybridization of bone sections showed that Dmp1 expression was elevated in Kl−/− mice. (B and C) mRNA expression of Dmp1 and Mgp in osteoblasts was quantified by qPCR analysis. Calvarial osteoblasts were isolated and cultured in osteogenic medium for 2 weeks. *: p<0.05, **: p<0.01 vs WT; ##: p<0.01 vs Kl−/−. (TIF) [file pgen.1002726.s003.tif]

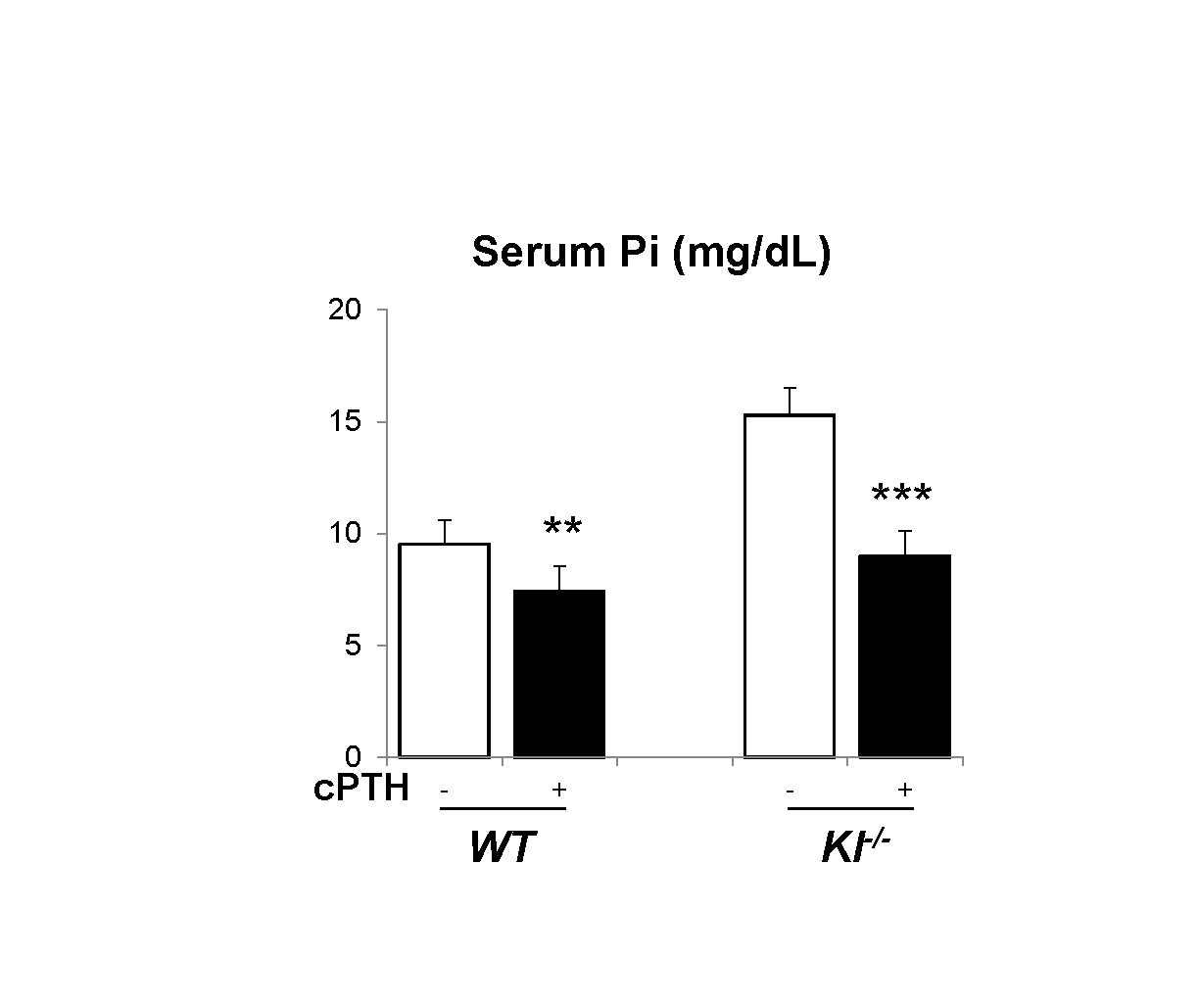

Supplement: Figure S4 — Serum phosphate measurements. PTH infusion significantly decreased serum phosphate levels in both WT and Kl−/− animals. **: p<0.01, ***: p<0.001vs vehicle controls. (TIF) [file pgen.1002726.s004.tif]

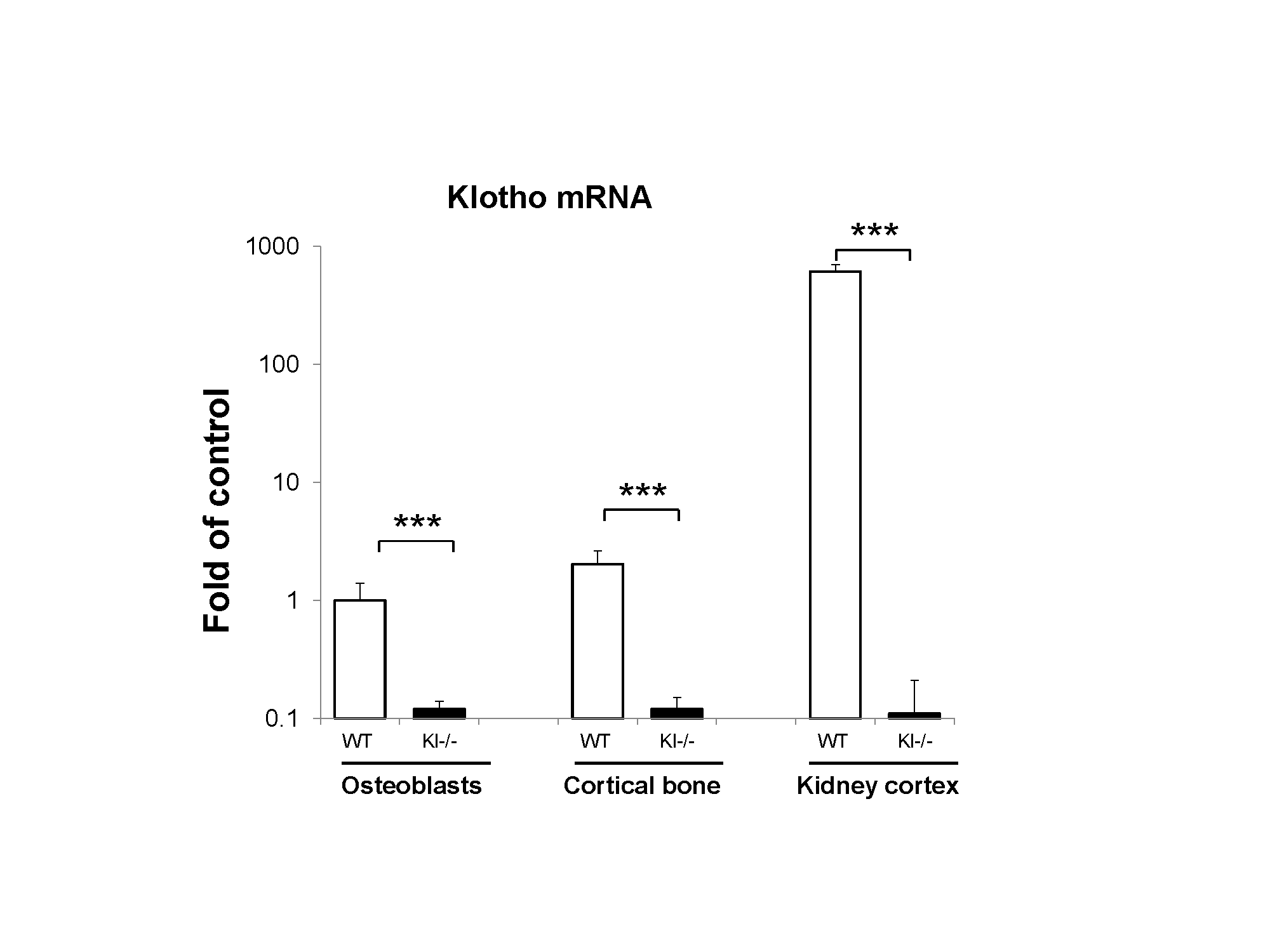

Supplement: Figure S5 — Gene expression of Klotho in bone and osteoblasts quantified by qPCR. Klotho expression was detected in the both cultured osteoblasts and cortical bones. However, it was much lower than that observed in the kidney. Tissues from Kl−/− mice were used as negative controls. ***: p<0.001. (TIF) [file pgen.1002726.s005.tif]
